# Interleukin 21-Armed EGFR-VHH-CAR-T Cell Therapy for the Treatment of Esophageal Squamous Cell Carcinoma

**DOI:** 10.3390/biomedicines13071598

**Published:** 2025-06-30

**Authors:** Chenglin Zhang, Yanyan Liu, Haoran Guo, Ying Peng, Lei Huang, Shuangshuang Lu, Zhimin Wang

**Affiliations:** 1 National Center for International Research in Cell and Gene Therapy, Sino-British Research Centre for Molecular Oncology, School of Basic Medical Sciences, Academy of Medical Sciences, Zhengzhou University, Zhengzhou 450052, China; zhangchenglin1995@gs.zzu.edu.cn (C.Z.);; 2 Department of Internal Medicine, Affiliated Cancer Hospital of Zhengzhou University & Henan Cancer Hospital, Zhengzhou 450052, China; yyliu@zzu.edu.cn; 3 Beijing An Zhen Hospital, Capital Medical University Affiliated Anzhen Hospital, Beijing 100029, China; 4 Translational and Clinical Research Institute, Faculty of Medical Sciences, Newcastle University, Newcastle NE1 7RU, UK; lei.huang@newcastle.ac.uk

**Keywords:** ESCC, EGFR, nanobody, CAR-T cells, human IL-21

## Abstract

**Background/Objectives:** Esophageal squamous cell carcinoma (ESCC) is a common form of esophageal cancer with a poor prognosis and limited treatment options. Epidermal growth factor receptor (EGFR), an overexpressed oncogenic gene in all ESCC patients, is an attractive target for developing therapies against ESCC. There is an extremely urgent need to develop immunotherapy tools targeting EGFR for the treatment of ESCC. **Methods:** In this study, we developed human Interleukin-21 (hIL-21)-armed, chimeric-antigen-receptor-modified T (CAR-T) cells targeting EGFR as a new therapeutic approach. The CAR contains a variable domain of the llama heavy chain of heavy-chain antibodies (VHHs), also known as nanobodies (Nbs), as a promising substitute for the commonly used single-chain variable fragment (ScFv) for CAR-T development. **Results:** We show that nanobody-derived, EGFR-targeting CAR-T cells specifically kill EGFR-positive esophageal cancer cells in vitro and in animal models. Human IL-21 expression in CAR-T cells further improved their expansion and antitumor ability and were observed to secrete more interferon-gamma (IFN-γ), tumor necrosis factor alpha (TNF-α), and Interleukin-2 (IL-2) when co-cultured with ESCC cell lines in vitro. More CD8^+^ CAR-T cells and CD3^+^CD8^+^CD45RO^+^CD62L^+^ central memory T cells were detected in CAR-T cells expressing hIL-21 cells. Notably, hIL-21-expressing CAR-T cells showed superior antitumor activity in vivo in a KYSE-150 xenograft mouse model. **Conclusions:** Our results show that hIL-21-armed, nanobody-derived, EGFR-specific CAR-T cell therapy is a highly promising option for treating ESCC patients.

## 1. Introduction

Esophageal cancer is among the top 10 most prevalent malignancies globally, characterized by high morbidity and mortality rates [1]. Esophageal adenocarcinoma (EAC) and esophageal squamous cell carcinoma (ESCC) are the two primary subtypes of esophageal cancer. Notably, China harbors over half of the world’s esophageal cancer patients, with ESCC constituting more than 90% of these cases [2]. ESCC is an aggressive disease with rapid progression and a high recurrence rate. The 5-year survival rates of ESCC patients are below 30%, emphasizing the need for advanced clinical research [3]. ESCC is typically treated with conventional chemotherapy, typically involving cisplatin and 5-fluorouracil (5-FU) (CF) [4,5]. However, the efficacy is limited and the prognosis is poor. Therefore, a more effective therapeutic strategy to treat ESCC is an unmet clinical need.

Epidermal growth factor receptor (EGFR) is a receptor tyrosine kinase abundantly expressed on ESCC cells and other epithelial-derived tumors with poor prognoses. EGFR can be targeted by CAR-T cell therapy [6]. The extracellular domain of EGFR protein consists of four domains: I, II, III, and IV. The binding of the EGFR ligand to EGFR extracellular domains I and III results in a conformational change in the structure of EGFR, exposing domain II, which was originally bound to domain IV, and subsequently dimerizing with another ligand-activated EGFR protein, resulting in the autophosphorylation of the EGFR protein [7]. EGFR is highly expressed in ESCC cells and has a low level of expression in normal epithelial cells. This difference makes EGFR a prime therapeutic target [8]. EGFR-targeted CAR-T cell therapy has been applied in clinical trials to treat various cancers, including triple-negative breast cancer, head and neck cancer, glioblastoma, and non-small cell lung cancer, and some of them have shown promising outcomes [9,10,11,12].

Nanobodies (Nbs), also named single variable domains on a heavy chain (VHHs), have been used in CAR development as an antigen recognition domain in place of the single-chain fragment variable (ScFv) [13]. Compared to ScFv, VHHs are more soluble and less prone to forming a multimeric complex on the cell membrane’s surface [14]. Additionally, the smaller size of VHHs allows for the integration of more genes into the vector to optimize T cell function [15]. Nbs were found and isolated from alpacas, and their immunogenicity is lower than that of traditional ScFv due to their small size and the development of humanized technology [16]. VHH-based CAR-T therapy has been used in targets including BCMA, vascular endothelial growth factor receptor 2 (VEGFR2), human epidermal growth factor receptor 2 (HER2), tumor-associated glycoprotein 72 (TAG-72), prostate-specific membrane antigen (PSMA), and Mucin 1 (MUC1) [13]. In particular, VHH-based CAR-T therapy that targets BCMA has been approved by the FDA for its potent tumor-suppressive function in treating multiple myeloma (MM), achieving an 88% overall response (OR) and a 68% complete response (CR) [17]. EGFR-targeting VHHs have been isolated and identified in previous studies [18,19,20]. However, there are few reports of the application of nanobody-based, EGFR-targeting CAR-T cell therapy.

Interleukin 21 (IL-21), a multifunctional cytokine from the common receptor gamma chain family, is known for its role in promoting CD8^+^ killer T cell proliferation, enhancing IFN-γ secretion [21,22], and preventing T cell exhaustion [23]. Unlike lL-2, lL-7, and lL-15, IL-21 selectively activates the signal transducer and activator of transcription 1 (STAT1) and 3 (STAT3) and slightly activates STAT5A or STAT5B, demonstrating its role in modulating T cell-mediated antitumor immunity [24]. Previous studies have demonstrated that IL-21 potentiates CAR-T cell efficacy through multiple mechanisms: enhancing the proliferative capacity, delaying cellular senescence, and inhibiting terminal differentiation into advanced memory phenotypes [25]. Furthermore, this cytokine promotes the tumor infiltration of CD19-specific CAR-T cells and subsequently induces tumor growth [26].

In this study, we used an EGFR-specific Nb, VHH clone 7D12 [20] as the antigen-targeting domain of CARs. We developed two types of nanobody-based CAR-T cells: 7D12-CAR-T cells (7D12-T) and 7D12-CAR-T cells expressing human IL-21 (7D12-T-IL21). We examined in vitro expansion, cytokine secretion, killing activity against the three ESCC cell lines with different EGFR expression levels, and in vivo tumor control in a xenograft model.

## 2. Materials and Methods

### 2.1. Cell Lines and Culture

Human ESCC cell lines, including KYSE-30 cells, KYSE-70 cells, KYSE-140 cells, KYSE-150 cells, KYSE-180 cells, KYSE-270 cells, KYSE-410 cells, KYSE-450 cells, and KYSE-510 cells, were obtained from local Biobank. The cells were cultured in an RPMI-1640 medium (GIBCO, Thermo Fisher Scientific, Waltham, MA, USA) with 10% fetal bovine serum (FBS, PAN). Human T cells were maintained in X-VIVO 15 media (Lonza, Basel, Switzerland) supplemented with 100 IU/mL human IL-2 (PeproTech, Cranbury, NJ, USA).

### 2.2. Generation of EGFR-Deficient ESCC Cell Lines

EGFR-deficient KYSE-30, KYSE-150, and KYSE-510 cell lines were generated using CRISPR-Cas9-based, site-directed mutagenesis, as described in a previous report [27]. The gRNA sequence for human EGFR is 5′-GTGGAGCCTCTTACACCCAG-3′, as described previously [28].

### 2.3. Real-Time Quantitative Reverse Transcription PCR

To extract RNA, Trizol was used, as described previously [29]. cDNA was obtained by reverse transcribing 100 ng of RNA using HiScript III All-in-one RT SuperMix Perfect for qPCR kit (Vazyme, Nanjing, China). Real-Time Quantitative PCR was performed using a ChamQ Universal SYBR qPCR Master Mix (Vazyme). The forward human EGFR qPCR primer is 5′-AGGCACGAGTAACAAGCTCAC-3′, and the reverse human EGFR qPCR primer is 5′-ATGAGGACATAACCAGCCACC-3′. The forward human GAPDH qPCR primer is 5′-GTCTCCTCTGACTTCAACAGCG-3′, and the reverse human GAPDH qPCR primer is 5′-ACCACCCTGTTGCTGTAGCCAA-3′.

### 2.4. Western Blot

Cells were lysed in an RIPA lysis buffer. The protein concentration in the lysate was measured using a BCA Protein Assay, as described previously [30]. The antibodies used for Western blotting included anti-GAPDH (Proteintech, Rosemont, IL, USA, Cat.#60004-128, dilution of 1:5000) and anti-human/mouse EGFR (Invitrogen, Carlsbad, CA, USA, Cat. #MA5-13070, dilution of 1:1000).

### 2.5. Isolation and Expansion of T Cells

Fully human PBMCs were obtained from Zhengzhou Central Blood Station and isolated via density gradient centrifugation, as previously described [31]. The procedures for all works using human material for research received ethical approval from the Ethics Committee of Zhengzhou University (License 17090000000224639). We confirm that all the methods were performed in accordance with the relevant guidelines and regulations and that informed consent was obtained. Human T cells were isolated from PBMC using anti-human CD3/CD28 Dynabeads (Gibco, Thermo Fisher Scientific, Waltham, MA, USA) and cultured in an X-VIVO 15 medium (LONZA) supplemented with 5% (*v*/*v*) fetal bovine serum, 100 IU/mL human IL-2 (Peprotech), and 1% penicillin–streptomycin.

### 2.6. Lentivirus Production and T Cell Transduction

Lentiviral vectors were produced in 293T cells. Culture supernatants containing lentiviral particles were harvested 48 and 72 h post transfection and filtrated through a 0.45 μm pore size membrane and then ultracentrifuged at 10,000× *g* for 16 h. The pellet containing the lentiviral particles was resuspended in an X-VIVO medium and kept frozen at −80 °C. The human CD3^+^ T cells were isolated and stimulated with anti-human CD3/CD28 beads (thermo-fisher 11161D). After 48 h of in vitro stimulation, 1 × 10^5^ T cells were seeded with the lentivirus at a multiplicity of infection (MOI) of 5 in a 24-well plate and centrifuged at 32 °C, 1200× *g* for 90 min. The transduction medium was replaced after another 4 h, and then the T cells were cultured with a fresh medium that was replenished every two days at a cell density of 3–5 × 10^5^ per mL.

### 2.7. Flow Cytometry Assays

The following antibodies were used: EGFR Monoclonal Antibody (H11) (Thermo-fisher, Waltham, MA, USA, Cat# MA5-13070); Alexa Fluor 488-labeled Goat Anti-Mouse IgG (H + L) (Beyotime, Shanghai, China); and MonoRab™ Rabbit Anti-Camelid VHH Cocktail [iFluor 488] (Genscript, Piscataway, NJ, USA). Human T cell surface markers were detected using an FITC-conjugated mouse anti-human CD3 (BioLegend, San Diego, CA, USA), Alexa Fluor 700-conjugated mouse anti-human CD4 (BioLegend), APC-conjugated mouse anti-human CD4 (BioLegend), APC-conjugated mouse anti-human CD8 (BioLegend), Percp/cy5.5-conjugated mouse anti-human CD8 (BioLegend), Brilliant Violet 650-conjugated mouse anti-human CD62L (BioLegend), and Brilliant Violet 421-conjugated mouse anti-human CD45RO (BioLegend). To detect the surface expression of EGFR on 293T cells transiently transfected with mouse EGFR or human EGFR, we used our in-house-produced 7D12-mouse-IgG-Fc protein as the primary antibody at a concentration of 40 μg/mL, followed by the secondary antibody, FITC anti-mouse IgG antibody (BioLegend).

### 2.8. Surface Plasmon Resonance (SPR) Assay

The binding kinetics and affinity of the interaction between 7D12-mouse-IgG-Fc and mouse EGFR His-Tag protein were measured using BIAcore. Additionally, 1 × HBS-EP+ buffer (10 mM HEPES, 150 mM NaCl, 3 mM EDTA, 0.05% surfactant P20, pH 7.4) was used as the running buffer for the experimental operation. Mouse EGFR His-Tag protein at one concentration per injection cycle was measured for association and dissociation with 7D12-mouse-IgG-Fc protein. Flow cell 1 was used as a blank reference channel. At a flow rate of 10 μL/min, the 7D12-mouse-IgG-Fc protein was diluted to 5 μg/mL with the running buffer and captured on flow cell 2 of the CM5 chip for 120 s, and the capture level was about 1620RU. At a flow rate of 30 μL/min, the diluted mouse EGFR His-Tag protein was sequentially injected into flow cells 1 and 2 at a concentration of 312.5–10,000 nM (double dilution); the association time was 60 s, and the dissociation time was 90 s. CM5 chip regeneration was completed through the injection of 10 mΜ glycine, pH 1.5, at a flow rate of 30 µL/min for 30 s. The experimental temperature was 25 °C. Using T200 Evaluation Software (version 3.2), mouse EGFR His-Tag protein, and 7D12-mouse-IgG-Fc protein were analyzed by a 1:1 binding mode, where ka is the association rate constant, kd is the dissociation rate constant, and KD is the affinity constant.

### 2.9. Cytotoxicity Assays

The Cytotoxicity LDH Assay Kit-WST (DOJINDO, Shanghai, China) was used to detect the LDH secretion of KYSE cell lines that were co-cultured with CAR-T cells or UTD T cells at different ratios (T cells: target cells = 2:1, 4:1, 8:1, 16:1, and 32:1). After 16 h, the culture supernatants were collected and detected by using an LDH release assay, a human IFN-γ ELISA kit (MultiSciences, Hangzhou, China), a human TNF-α ELISA kit (MultiSciences), a human IL-2 ELISA kit (MultiSciences), and a human IL-10 ELISA kit (MultiSciences), respectively. Each experiment was performed three times with three different donors.

### 2.10. ELISA Assay

The human IL-21 ELISA Kit (Invitrogen, CA, USA) was used to measure IL-21 concentrations. Briefly, CAR-T cells and control T cells were cultured in 1 mL of X-VIVO medium with 5% (*v*/*v*) fetal bovine serum. Cell culture supernatants were collected and assayed at 24 h. The detection of hIL-21 was performed using the human IL-21 uncoated ELISA kit, according to the manufacturer’s instructions.

### 2.11. Xenograft Tumor Models

In this experiment, 6-week-old NSG male mice were purchased from Jiangsu Cyagen Biosciences Co., Ltd., Suzhou, China. All the animal study ethics were approved by Zhengzhou V3Biotech (Reference number: V3A02023000009). All the methods used in the animal study were reported and carried out in accordance with the ARRIVE guidelines and the institutional guidelines outlined by Zhengzhou V3Biotech. For KYSE-150 xenograft tumor models, 6-week-old NSG mice were inoculated s.c. with 5 × 10^6^ KYSE-150 cells on the right flank. When the tumor size was ~100 mm^3^ after approximately 7 days, the mice were randomized into 4 groups (6 mice in each group), and each mouse was injected through the tail vein (i.v.) with 5 × 10^6^ T cells in 100 μL of the control PBS; the groups of mice received either the PBS alone, PBS containing control T cells, PBS containing 7D12 T cells, or PBS containing 7D12-T-hIL-21 cells. The tumor size was detected every two days until the tumor size reached 1500 mm^3^. At the end of the experiment, the mice were euthanized.

### 2.12. Statistical Analysis

The statistical analysis was performed using GraphPad Prism 6 and IBM SPSS statistical software (version 19.0). A Student’s *t*-test was utilized to compare differences between two groups, while a one-way ANOVA was used to analyze differences among multiple groups. Additionally, a Kaplan–Meier survival analysis was employed to assess differences in survival. Statistical significance was considered when the *p*-values were at or below 0.05.

## 3. Results

### 3.1. EGFR Is Upregulated in ESCC and Associated with Poor Prognosis

**Figure 1:**
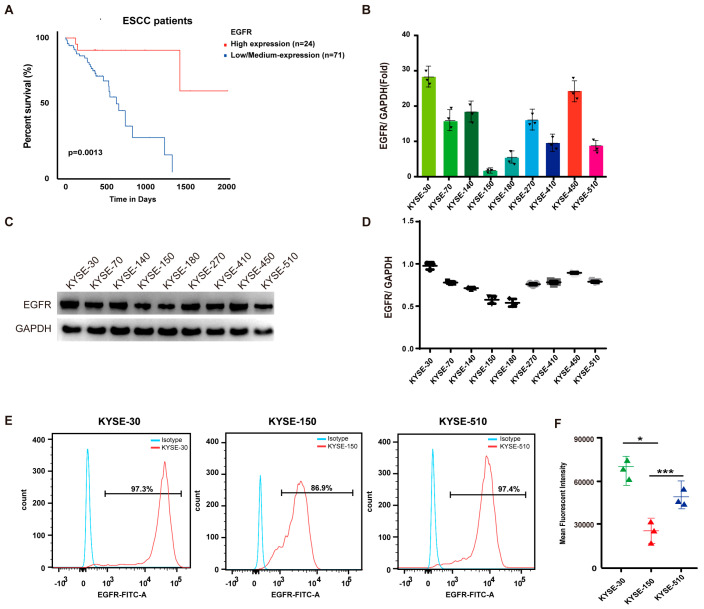
EGFR is upregulated in ESCC with predicted poor prognosis. (**A**) Kaplan–Meier analysis of association of EGFR expression with OS in ESCC patients. (**B**) qPCR analysis of mRNA levels of EGFR in ESCC cell lines. (**C**) Western blot analysis of EGFR protein levels in ESCC cell lines, including KYSE-30, KYSE-70, KYSE-140, KYSE-150, KYSE-180, KYSE-270, KYSE-410, KYSE-450, and KYSE-510. (**D**) Quantification of expression of EGFR in all nine ESCC cell lines (n = 3 per group). (**E**) Flow cytometry analysis of EGFR expression in KYSE-30 cells, KYSE-150 cells, and KYSE-510 cells. (**F**) Mean fluorescence intensity of staining for EGFR expressions in KYSE-30 cells, KYSE-150 cells, and KYSE-510 cells. Statistical analysis was performed using two-tailed, unpaired Student’s *t*-test. * *p* < 0.05; *** *p* < 0.001.

We analyzed how EGFR expression impacted the overall survival of ESCC patients using the web tool from the University of Alabama at Birmingham cancer database (UALCAN). Employing the quartile method for the statistical analysis, we sorted the samples with the top 25% expression levels into the high-expression group and others into the medium-to-low expression group. Subsequently, a Cox regression analysis was performed to assess survival differences. Significant survival differences were observed between the EGFR high-expression group (including 24 ESCC patients in the top quadrant) and the EGFR low/medium-expression group (including 71 ESCC patients in the lower third quadrant) amongst all the ESCC patients in the dataset (Figure 1A). Then, we screened the nine locally established ESCC cell lines (KYSE-30, KYSE-70, KYSE-140, KYSE-150, KYSE-180, KYSE-270, KYSE-410, KYSE-450, and KYSE-510) for their EGFR expression level using qPCR (Figure 1B), and we established that KYSE-30 and KYSE-150 have the highest and lowest levels of EGFR mRNA, respectively. This result was further confirmed by Western blotting (Figure 1C,D). Our findings reveal a high prevalence of EGFR mRNA and protein expression in ESCC cells. We then selected three cell lines with different levels of EGFR expression, namely KYSE-30 (high expression), KYSE-510 (intermediate expression), and KYSE-150 (low expression), to represent all the scenarios of the EGFR level to test our therapy. The EGFR expression levels in these three cell lines were further tested using flow cytometry and confirmed using the mean fluorescence intensity (Figure 1E,F). Therefore, KYSE-30, KYSE-150, and KYSE-510 were used for the subsequent experiments based on their distinct EGFR expression levels.

### 3.2. EGFR-VHH-7D12 Nanobody Effectively Identifies EGFR

**Figure 2:**
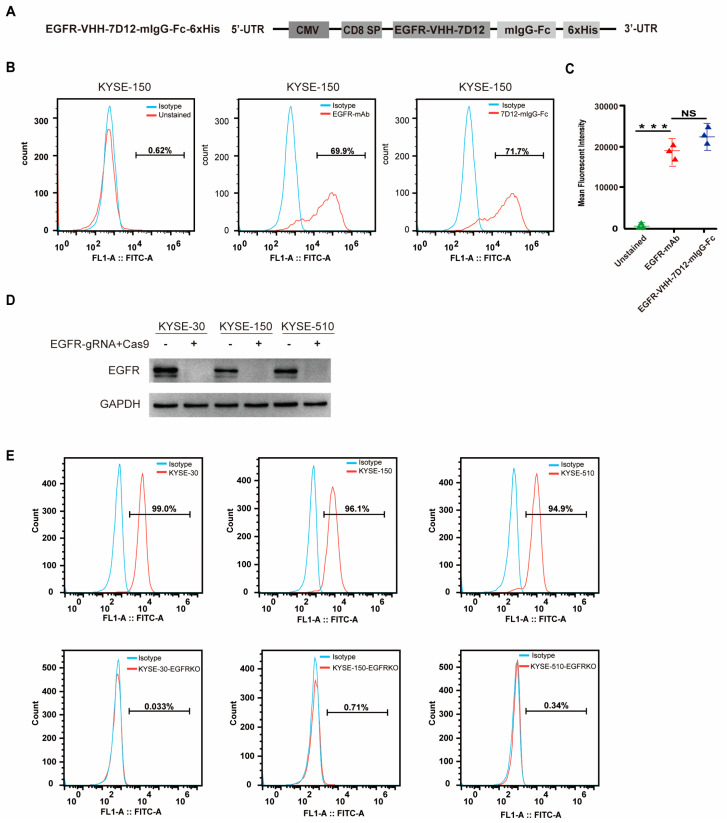
EGFR-VHH-7D12 nanobody effectively recognizes EGFR. (**A**) Schematic representation of plasmid construct of EGFR-VHH-7D12-mIgG-Fc-6xHis used for generation of 7D12-mIgG-Fc infusion protein. (**B**) Flow cytometry analysis of EGFR expression in KYSE-150 cells with 7D12-mIgG-Fc protein and commercial monoclonal mAb. (**C**) Mean fluorescence intensity of EGFR in KYSE-30 cells detected using PBS blank control, EGFR mAb, and EGFR-VHH-7D12-mIgG-Fc. (**D**) Expression of EGFR in ESCC cell lines and EGFR-knockout ESCC cell lines, including KYSE-30, KYSE-150, and KYSE-510, detected using Western blotting. (**E**) EGFR-VHH-7D12-mouse IgG Fc fusion protein was used in flow cytometry to detect expression of EGFR in WT and EGFR-knockout ESCC cell lines (Red). Statistical analysis was performed using two-tailed, unpaired Student’s *t*-test; NS: no statistical significance. *** *p* < 0.001.

The Nb EGFR-VHH-7D12, known for its ability to specifically target the membrane protein EGFR, has been increasingly utilized in immunotherapy applications in recent years [19]. To confirm the specificity of EGFR-VHH-7D12, we designed an EGFR-VHH-7D12-mIgG-Fc-6xHis plasmid to generate 7D12-mIgG-Fc protein (Figure 2A). Flow cytometry showed that our 7D12-mIgG-Fc protein produced similar staining to a commercialized monoclonal antibody (EGFR-mAb) regarding both the mean fluorescent intensity and the percentage of positive cells (Figure 2B,C). To evaluate the specificity of 7D12-VHH in recognizing EGFR, the EGFR-VHH-7D12-mIgG-Fc fusion protein was tested with the KYSE cell lines, with the EGFR gene being knocked out using Crispr-Cas9 technology. The 7D12-VHH can specifically recognize the wild type of KYSE-30, KYSE-150, and KYSE-510 cell lines but not EGFR-deficient cell lines (Figure 2D,E).

### 3.3. Generation of hIL-21-Armed VHH-7D12 Nanobody CAR-T Cells

**Figure 3:**
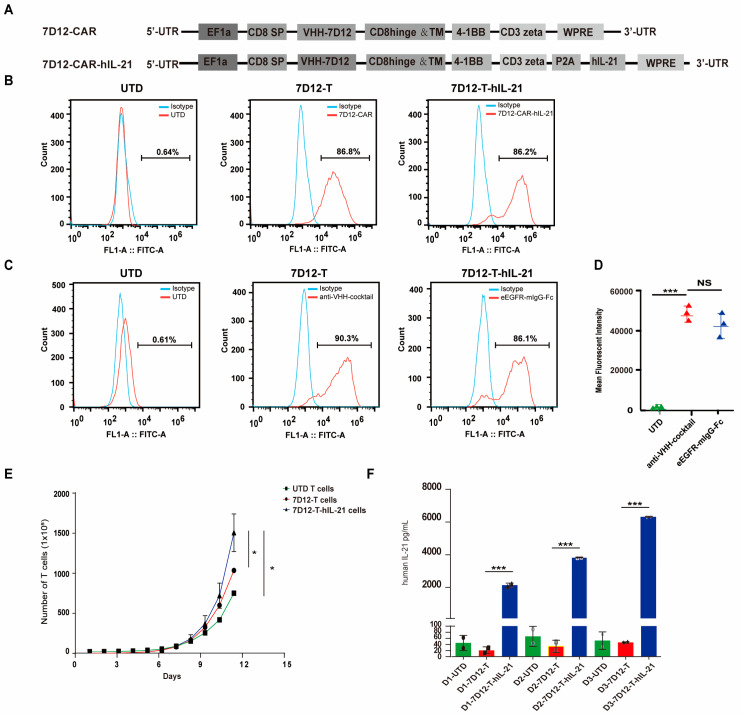
Generation of hIL-21-armed 7D12-T cells. (**A**) Schematic representations of constructs of 7D12-CAR and 7D12-CAR-hIL-21. (**B**) Flow cytometry was performed to detect expression of 7D12-CAR in UTD T cells, 7D12-T cells, and 7D12-T-hIL-21 cells by anti-VHH-FITC antibody. (**C**) Flow cytometry analysis was conducted to compare detection efficiency between EGFR-mIgG-Fc protein (eEGFR-mIgG-Fc) and anti-VHH-FITC (anti-VHH-cocktail) for 7D12-T cells. (**D**) Mean fluorescence intensity analysis and statistical summary for results in panel C. (**E**) In vitro expansion rates of UTD T, 7D12-T cells, and 7D12-T-hIL-21 cells. (**F**) IL-21 levels in supernatant from cultured UTD T cells, 7D12-T cells, and 7D12-T-hIL-21 cells 7 days after transfection of CAR-lentiviral particles. Data analysis was repeated using T cells from three different donors. Statistical analysis was performed using two-tailed, unpaired Student’s *t*-test. NS: no statistical significance. *** *p* < 0.001.

VHH-7D12 was employed as an alternative to ScFvs to construct the 7D12-CAR, integrating VHH-7D12, CD8 hinge and transmembrane domains, 4-1BB, and the CD3ζ signaling domain. IL-21 promotes the proliferation, survival, differentiation, and function of activated T cells, which can also enhance the antitumor ability of CD8^+^ T cells and natural killer cells [32]. Therefore, we introduced human IL-21 into the 7D12-CAR construct. The hIL-21 enhancement was engineered by appending a P2A peptide, a self-cleavage peptide, and human IL-21 to the C-terminus of 7D12-CAR (Figure 3A). The use of P2A allows the two transcripts to share the same promoter, therefore increasing the efficacy in lentiviral particle packaging. We generated lentiviral particles to deliver the CARs into preactivated human T cells. To determine gene delivery efficacy, anti-VHH-FITC was used to detect the expression of CAR on the surface of T cells by flow cytometry (Figure 3B). We further confirmed CAR specificity by using the soluble EGFR-mIgG-Fc fusion protein as a bait protein to detect the expression of 7D12-CAR on the surface of 7D12-T cells. Flow cytometry shows that both the anti-VHH-FITC antibody and EGFR-mIgG-Fc can effectively detect 7D12-CAR-T cells (Figure 3C,D) with similar sensitivity. The 7D12-CAR-hIL-21-T cells exhibited a better proliferation capacity compared with the 7D12-CAR-T cell group and the UTD T cell group (Figure 3E). hIL-21 was detected in the supernatant from the 7D12-CAR-hIL-21-T cell culture but not from the 7D12-CAR-T cell culture nor the control T cell cultures from any of the three donors (Figure 3F). No significant changes in exhaustion markers were detected in either the 7D12-T cells or the 7D12-T-hIL21 cells after in vitro culturing (Supplementary Figure S3). These results collectively demonstrate the target specificity of 7D12-CAR and successful hIL-21 expression in armed 7D12-CAR-T cells.

### 3.4. Influence of hIL-21 on the Phenotype of 7D12-T Cells

**Figure 4:**
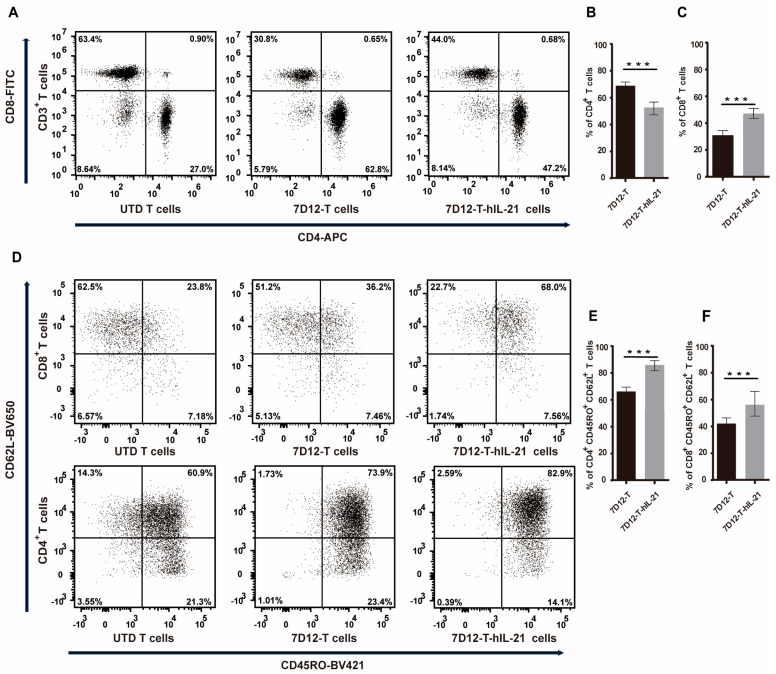
Influence of hIL-21 on CAR-T cells’ phenotypes. (**A**) Dot plot indicates proportions of CD4^+^ and CD8^+^ T cell subsets in 7D12-T and 7D12-T-hIL-21 CAR cells after culturing for 7 days in vitro. Histogram indicates decrease in proportion of CD4^+^ (**B**) and increase in CD8^+^ (**C**) T cells. (**D**) Phenotyping of T cells indicates increase in CD45RO^+^CD62L^+^ Tcm cells in CD8^+^ (up) and CD4^+^ (below) T cells in 7D12-T and 7D12-T-hIL-21 CAR-T cells. Histogram shows proportion of CD45RO^+^CD62L^+^ T cells in CD4^+^ T cells (**E**) and CD8^+^ T cells (**F**). Statistical analysis was performed using two-tailed, unpaired Student’s *t*-test; *** *p* < 0.001.

IL-21 has been documented to significantly improve the T cell condition in cultures [33]. Our study elucidates its impact on the CAR-T cell immune memory phenotype. After 11 days in culture, 7D12-T-hIL-21 cells exhibited a decrease in the CD4^+^ (50% vs. 63% *p* < 0.001) and an increase in the CD8^+^ T cell proportion (41% vs. 28% *p* < 0.001) compared to 7D12-T cells without hIL-21 arming (Figure 4A–C). This suggests that IL-21 plays a role in regulating the CD4^+^ and/or CD8^+^ T cell growth within the CAR-T cell milieu. Moreover, we investigated the memory T cell subsets elicited amongst 7D12-T or 7D12-T-hIL-21 cells. The latter contained a higher proportion of CD3^+^/CD45RO^+^CD62L^+^ central memory T (Tcm) cells in both CD4^+^ (84% vs. 68% *p* < 0.01) and CD8^+^ (56% vs. 44%, *p* < 0.01) T cells compared to 7D12-T cells (Figure 4D–F). These results suggest that IL-21 skews CAR-T cells towards a more cytotoxic Tcm phenotype.

### 3.5. In Vitro Cytotoxicity of CAR-T Cells Against ESCC Cell Lines

**Figure 5:**
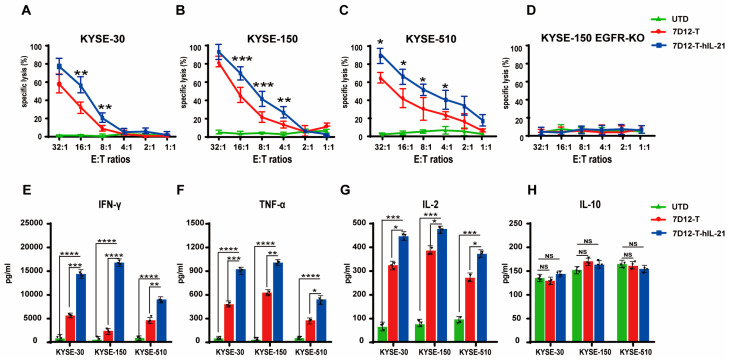
In vitro cytotoxicity of EGFR-CAR-T cells against ESCC cell lines. (**A**–**D**) Lactate dehydrogenase (LDH) release assay demonstrating cytotoxicity of UTD T cells, 7D12-T cells, and 7D12-T-hIL-21 cells when co-cultured with (**A**) KYSE-30 cells, (**B**) KYSE-150 cells, (**C**) KYSE-510 cells, and (**D**) KYSE-150 EGFR-KO cells. Cells were incubated at various effector-to-target (E/T) ratios at 37 °C for 16 h (n = 3 per group). (**E**–**H**) ELISA results measuring IFN-γ (**E**), TNF-α (**F**), IL-2 (**G**), and IL-10 (**H**) in culture supernatant after 16 h co-culture of CAR-T cells with ESCC cells at E/T ratio of 16:1 at 37 °C. Statistical analysis was performed using two-tailed, unpaired Student’s *t*-test; NS, not significant; * *p* < 0.05, ** *p* < 0.01, *** *p* < 0.001, and **** *p* < 0.0001.

EGFR is highly expressed in esophageal squamous cell carcinomas (ESCCs), highlighting its potential as a therapeutic target [34]. To evaluate the antitumor efficacy of 7D12-T and 7D12-T-hIL-21 cells, we utilized three ECSS cell lines with different levels (high, intermediate, and low) of EGFR expression as targets, namely KYSE-30 cells, KYSE-150 cells, and KYSE-510 cells, respectively, and we genetically engineered KYSE-150 to be deficient in EGFR cells as a negative control. Both 7D12-T and 7D12-T-hIL-21 cells demonstrated specific and potent cancer-specific cytotoxicity against the KYSE-30, KYSE-150, and KYSE-510 cell lines, but not against the negative control cells (Figure 5A–D). The 7D12-T-hIL-21 cells outperformed the unarmed 7D12-CAR-T cells in killing all three ESCC cell lines, regardless of the EGFR expression level, underlining their superior antitumor efficacy. Furthermore, we assessed the level of secreted cytokines, including IFN-γ, TNF-a, IL-2, and IL-10, produced from the co-culture of CAR-T cells with tumor cells at an effector-to-target (E:T) ratio of 16:1. The 7D12-T-hIL-21 cells secreted significantly higher levels of IFN-γ, TNF-α, and IL-2 compared to the 7D1-T cells (Figure 5E–G). However, the expression level of hIL-10 remained low and was not affected by the presence of hIL-21 (Figure 5H). These findings indicate the robust and consistent antitumor efficacy of nanobody-based CAR-T cells, regardless of the expression levels of their antigens, and show that hIL-21 augments the antitumor potency of CAR-T cells.

### 3.6. Efficacy of EGFR-CAR-T Cells in ESCC Cell Mouse Xenograft Models

**Figure 6:**
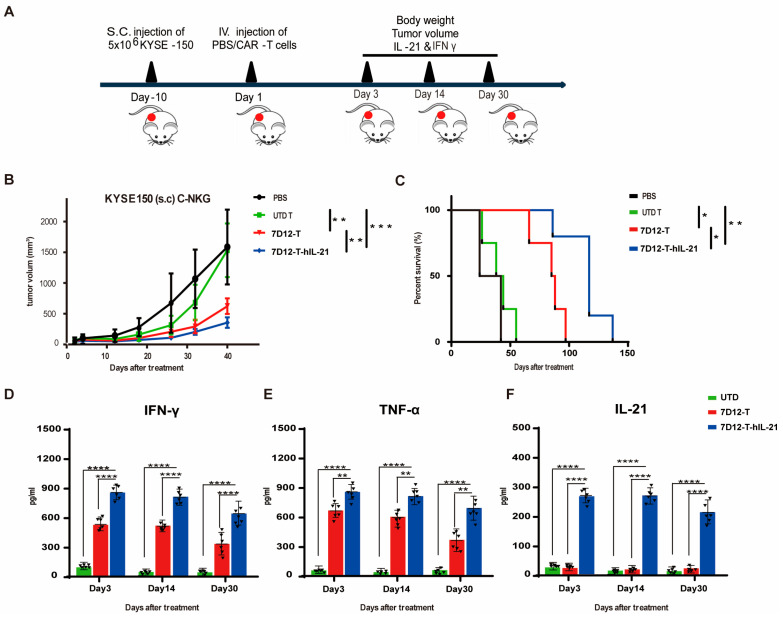
Therapeutic efficacy of 7D12-T (-hIL-21) cells in ESCC mouse xenograft models. (**A**) Schematic representation of experimental setup for evaluating efficacy of 7D12-T (-hIL21) T cell therapy in vivo. (**B**) Tumor growth was plotted as volume measured twice per week. (**C**) Kaplan–Meier (KM) plot of mice’s overall survival rates in each group, providing insights into potential life-prolonging effects of CAR-T cell therapies. (**D**) Presence of human cytokines, including IFN-γ (**D**), TNF-α (**E**), and IL21 (**F**), in serum of experimental mice measured using ELISA. Data are presented as mean ± SEM, with statistical significance denoted as * *p* < 0.05, ** *p* < 0.01, *** *p* < 0.001, and **** *p* < 0.0001.

We then evaluated the therapeutic potential of 7D12-T cells and 7D12-T-hIL-21 cells against ESCC mouse xenograft models. NSG mice were subcutaneously inoculated with 5.0 × 10^6^ KYSE-150, the cells with low EGFR expression. Upon the tumor volumes reaching approximately 100 mm^3^, the mice were intravenously injected with 5.0 × 10^6^ 7D12-T cells, 7D12-T-hIL21 cells, or control (UTD) T cells. An amount of 100 μL of phosphate-buffered saline (PBS) was injected into an additional group as a vehicle control (Figure 6A). The mice treated with either the 7D12-T or the 7D12-T-hIL21 cells significantly slowed tumor growth (Figure 6B) and enhanced survival rates (Figure 6C) in comparison to the control treatments (UTD T cells and PBS). Furthermore, the 7D12-T-hIL-21 cells displayed superior tumor control and mouse survival in comparison to the unarmed 7D12-T cells (Figure 6B,C). The levels of the cytokines, including IFN-γ (Figure 6D) and TNF-α (Figure 6E), were significantly higher in the mice treated with the 7D12-T or the 7D12-T-hIL21 cells than the control mice, and the mice treated with the 7D12-T-hIL21 cells elicited the highest level of cytokines (Figure 6D,E) in the peripheral blood. hIL-21 was exclusively detected in the mice treated with the 7D12-T-hIL-21 cells (Figure 6F). These results demonstrate that our VHH-based CAR-T cell therapy is highly effective in curtailing the growth of subcutaneous tumors in the xenograft model even when its target, EGFR expression, is low. Arming 7D12-T cells with hIL-21 further enhanced therapeutic efficacy.

### 3.7. Safety Profile of EGFR-CAR-T Cells in ESCC Cell Mouse Xenograft Models

EGFR is a tumor-associated antigen that is also expressed in normal tissue, although at a substantially lower level; therefore, evaluating the on-target, off-tumor (OTOT) toxicity associated with 7D12-T cell therapy is imperative, especially for hIL21-armed 7D12-T cells [31]. To uncover whether mouse models are suitable for investigating OTOT toxicity, we tested the ability of VHH-7D12 to bind to mouse EGFR. For the first time, we determined that 7D12-mIgG-Fc recognized both human and murine EGFR using Western blotting and flow cytometry (Supplementary Figure S1A–C). In addition, we also measured the affinity between VHH-7D12 and murine EGFR using surface plasma resonance technology and determined that the dissociation constant (KD) of the binding between the 7D12-mIgG-Fc fusion protein and mouse EGFR was 2.9 µM (Supplementary Figure S2). It was previously reported that the dissociation constant of 7D12VHH for human EGFR is 219 ± 20 (279 ± 19) nM [20].

**Figure 7:**
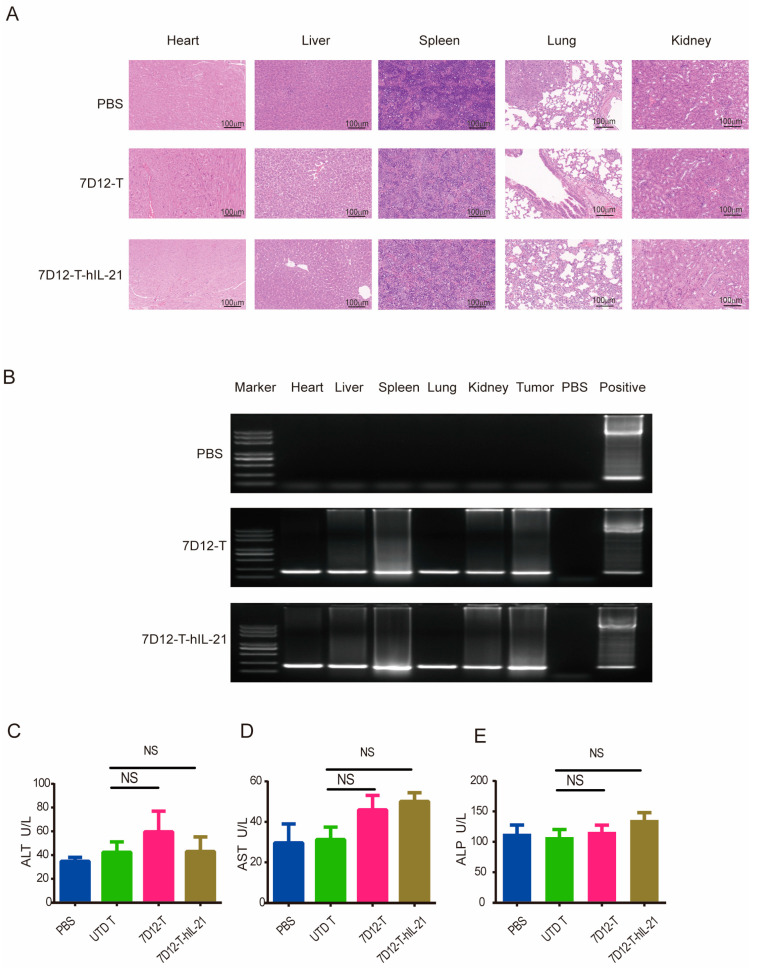
Assessing the safety of EGFR-CAR-T cells in ESCC cell mouse xenograft models. (**A**) Hematoxylin and eosin (H&E) staining was performed on major organ tissues (heart, liver, spleen, lung, and kidney) from mice treated with 7D12-T cells, 7D12-T-hIL21 cells, or a PBS control. Scale bar: 100 mm. (**B**) A PCR analysis was performed for the detection of residual genomic DNA from CAR-T cells in the heart, liver, spleen, lung, and kidney tissues. Levels of liver enzymes, including alanine aminotransferase (ALT) (**C**), aspartate aminotransferase (AST) (**D**), and alkaline phosphatase (ALP) (**E**), in the serum were measured to evaluate liver function as an indicator for any potential hepatotoxicity associated with CAR-T cell therapy. NS: no statistical significance.

Our investigation focused on potential normal tissue damage in mouse xenograft models treated with 7D12-T cells or 7D12-hIL-21-T cells in comparison to those treated with the controls. A histopathology analysis across all the groups revealed no significant tissue damage in critical organs, including the heart, liver, spleen, lung, and kidney (Figure 7A), despite the low level of EGFR expression in some of these tissues [35,36,37]. We further examined the potential infiltration of CAR-T cells into normal tissues after the treatment. Genomic DNA extracted from major organs was subjected to PCR amplification using WPRE-specific primers. WPRE sequences were detected in the 7D12-T and 7D12-T-hIL-21 cell treatment groups but not in the control group (Figure 7B). While immunohistochemistry did not indicate overt tissue damage, the presence of CAR-T cells in various tissues suggests a potential OTOT toxicity risk. Additionally, we assessed liver function by measuring the levels of liver enzymes (ALT, AST, and ALP) to identify signs of liver damage. The results show no significant increase in the liver enzyme levels in the mice treated with CAR-T cells (Figure 7C).

These findings collectively suggest that 7D12-T cell therapy does not induce significant tissue damage, regardless of whether it is armed with hIL-21, despite evidence of CAR-T cell presence in non-target tissues. The safety assessment underscores the potential of 7D12-based CAR-T cell therapy for clinical application, highlighting its favorable safety profile in the context of ESCC treatment.

## 4. Discussion

In this study, we engineered and integrated an EGFR-specific Nb, VHH-7D12, as the antigen recognition domain into the structure of CARs to generate 7D12-T cells and further generated hIL-21-armed 7D12-T (7D12-T-hIL-21) cells to enhance their antitumor activity. High EGFR expression is observed in 50–70% of ESCC patients and is associated with tumor invasiveness and patients’ poor survival [38,39,40]. Our findings indicate that both 7D12-T cells and 7D12-T-hIL-21 cells are effective in killing ESCC cells in vitro and inhibiting tumor growth in vivo in a xenograft model. Compared to 7D12-T cells, 7D12-T-hIL-21 cells are superior in terms of their in vitro expansion, ability to form long-lived central memory T cells, and antitumor potency, both in vitro and in vivo. Our results indicate the potential of Nb-based CAR-T cells, especially those armed with hIL-21, as a promising treatment for ESCC, offering a new immunotherapeutic strategy for this aggressive cancer.

CAR-T cells have demonstrated remarkable efficacy in identifying and destroying tumor cells, particularly in hematological malignancies [41]. However, their antitumor capability in solid tumors, including ESCC, remains to be demonstrated [42]. Heavy-chain antibodies (HCAbs) were isolated in the peripheral blood of dromedary camels. The heavy-chain variable region domain of HCAbs can be genetically cloned and engineered to create VHH antibodies, also known as nanobodies [43]. Compared to ScFvs, Nbs are structurally more stable and smaller in size and exhibit a comparable recognizing and binding ability with lower immunogenicity [13]. Therefore, the use of Nbs to replace the traditional ScFvs as the extracellular antigen recognition region of CARs is a plausible approach to enhance antitumor potency.

The utilization of single-domain antibodies, such as the 7D12 nanobody, in chimeric antigen receptor (CAR) design confers several distinct advantages over conventional scFv-based architectures. First, VHHs exhibit an inherently low immunogenicity due to their human-like framework sequences, significantly reducing the risk of anti-drug antibody (ADA) responses that can compromise CAR-T cell persistence [14]. Second, their compact size (<15 kDa) substantially enhances their viral packaging capacity during lentiviral transduction, enabling the more efficient delivery of complex genetic payloads. This expanded cargo space readily accommodates the integration of supplementary transgenes, such as cytokines, chemokine receptors, or safety switches, to augment their therapeutic functionality [14].

Critically, the superior hydrophobicity profile of VHHs promotes the proper folding and solvent exposure of antigen-binding domains, ensuring stable extracellular recognition structures essential for target engagement. This structural robustness provides a critical foundation for developing next-generation, bispecific VHH-CARs, where dual targeting modules can be incorporated without exceeding viral packaging limits. Such bispecific designs enhance tumor recognition specificity through avidity effects while mitigating antigen escape [44]. Furthermore, the minimal steric footprint of VHHs allows for the streamlined production of dual-VHH-CAR lentiviral vectors, overcoming the manufacturing bottlenecks associated with bulkier multi-chain constructs. Collectively, these attributes position VHHs as an enabling platform for advanced CAR-T engineering.

CarvyktiTM, a BCMA-targeted, Nb-based CAR-T therapy, is the first FDA-approved, commercial, Nb-based CAR-T product, signaling the strong potential of Nbs to be used in CAR-T cell therapy [45]. However, Carvykti is designed for multiple myeloma, and CAR-T therapy for solid tumors, especially for ESCC, is lacking. In this study, novel Nb-based CAR-T cells targeting EGFR were developed based on the VHH sequence 7D12 (VHH-7D12). This specific VHH has been applied in photodynamic therapy (PDT) by conjugation with a photosensitizing chemical substance (7D12-PS) [46]. The 7D12-PS elicited robust anti-tumor potency and low normal tissue toxicity. We designed and produced a 7D12-mIgG-Fc fusion protein and successfully utilized it as a primary antibody for flow cytometry at varying concentrations. On the other hand, we generated an EGFR-mIgG-Fc fusion protein to detect the expression of VHH-7D12 in our 7D12-T cells and 7D12-T-hIL-21 cells. These findings show that VHH-7D12 is structurally intact, acting as a specific antigen recognition domain in CAR-T cell therapy.

The EGFR is commonly present on epithelial-derived tissue cell membranes [34,47]. EGFR activation, through ligand binding and subsequent dimerization, initiates signaling cascades that promote cell proliferation, angiogenesis, invasion, metastasis, and the inhibition of cell death [48]. Mutation in the EGFR gene is less common, making targeted therapies using receptor tyrosine kinases inhibitors (TKRIs) less likely to be effective in treating ESCC (PMID: 23426935). ScFv-based, EGFR-targeted CAR-T cells have shown some efficacy in various types of solid tumors, including triple-negative breast cancer (TNBC), non-small cell lung cancer (NSCLC), multiple glioblastoma (GBM), and ESCC [49,50,51,52]. In our research, therapies using Nb-based 7D12-T cells and 7D12-T-hIL-21 cells resulted in better clinical outcomes, as reflected by both a significantly slower tumor growth rate and longer survival in a preclinical mouse xenograft model, when treating established tumors. Our results suggest that Nbs might be a superior choice over ScFvs in generating a CAR that targets solid tumors.

Interleukin-21 (IL-21), produced by various immune cells, including activated CD4^+^ T cells and natural killer T cells, belongs to the common cytokine receptor g chain cytokine family. IL-21 promotes T cell proliferation, survival, and differentiation and maintains the function of active T cells, therefore enhancing the antitumor ability of CD8^+^ T cells and natural killer cells [46]. IL-21 was also reported to be capable of re-activating the exhausted cytotoxicity of CD8^+^ T cells that reside in the tumor microenvironment [53]. CAR-T cells expressing hIL-21 (IL-21 armed) showed better antitumor activity compared to their non-armed counterparts [54]. Additionally, IL-21 was reported to inhibit the development of regulatory T (Treg) cells [55]. IL21-armed CAR-T has been used in a clinical trial targeting several solid tumors expressing GPC3 antigen, including Liver Cancer, Rhabdomyosacoma, Malignant Rhabdoid Tumor, Liposarcoma, Wilms Tumor, and Yolk Sac Tumor (NCT04715191), but not ESCC. In our study, 7D12-T-hIL-21 cells outperformed 7D12-CAR-T cells in all the tested aspects. Higher percentages of CD3^+^CD45RO^+^CD62L^+^ central memory T cells were generated after the in vitro expansion of 7D12-T-hIL-21 cells in comparison to 7D21-T cells in both CD8^+^ and CD4^+^ T cells. Furthermore, hIL21-armed CAR-T cells exhibited enhanced cytokine secretion and improved antitumor effector function, regardless of the antigen load. These observations were also confirmed in vivo in mice grafted with human ESCC cells, showing effective tumor growth control and prolonged survival.

No significant normal tissue toxicity was detected in any of the mice treated with 7D12-T cells, regardless of whether they were armed with hIL-12 cells, according to the histopathology and the liver function tests. However, the widespread expression of EGFR in various tissues and organs is a key concern, especially for long-term consequences in CAR-T therapy for cancer. Studies using mouse models have many limitations, including the time span of the experiment, the number of cells transferred, and the co-morbidities that usually occur in cancer patients but not in specific pathogen-free (SPF), immune-deficient mice. Residues of the lentiviral vector sequence were detectable in our experiments, suggesting long-lasting CAR-T cell infiltration in non-malignant tissues, which might be quiescent in our study. Extensive tissue infiltration is a precondition for CAR cytotoxicity; thus, further evaluations of the probability and conditions for the occurrence of CAR cytotoxicity are required in subsequent experiments. Moreover, VHH-7D12 exhibits a higher affinity for human EGFR than for mouse EGFR. Therefore, murine models can only provide preliminary insights into the OTOT toxicity of VHH-7D12-CAR, representing a limitation common to many CAR-T studies conducted in mice. The further assessment of its OTOT toxicity will require subsequent clinical trials to clarify the potential risk.

## 5. Conclusions

In this study, the EGFR-specific nanobody VHH-7D12 was used to design a CAR-T cell therapy for solid tumors. The 7D12-T cells and the 7D12-T-hIL-21 cells exhibited effective antitumor abilities in vitro and in vivo without severe normal tissue damage. Arming 7D12-T cells by incorporating the hIL-21 coding sequence into the CAR vector improved T cell proliferation and antitumor function. The 7D12-T-hIL-21 cells secreted more cytokines, including IFN-γ, TNF-α, and hIL-2, when cultured with tumor cells and exhibited better antitumor effector functions. Our study suggests that using Nbs is a good option in designing CAR-T cells against solid tumors, and arming Nb-based CAR-T cells with hIL-21 further improves their antitumor efficacy.

## Data Availability

The data that support the findings of this study are available from the corresponding author upon reasonable request.

## Supplementary Materials

The following supporting information can be downloaded at: https://www.mdpi.com/article/10.3390/biomedicines13071598/s1, Supplementary Figure S1. The 7D12-VHH can recognize human and mouse EGFR. (A) The expression of EGFR in 293T cells and human EGFR plasmid-transfected and mouse EGFR plasmid-transfected 293T cells was detected using Western blotting (n = 3). (B) Flow cytometry was used to detect the expression of EGFR in 293T cells and human EGFR plasmid-transfected and mouse EGFR plasmid-transfected 293T cells. PEI (Polyethylenimin) was used as a transfection reagent, 1 µg of EGFR expression plasmid was transferred with 2 µL of PEI, and the flow cytometry of EGFR was detected 48 h later. Supplementary Figure S2. The affinity determination of mouse EGFR protein and 7D12-mouse IgG-Fc protein. The concentration range of mouse EGFR protein is 312.5–10,000 nM, and the measured parameters include Ka (association constant), Kd (dissociation rate constant), and KD (equilibrium dissociation constant). Supplementary Figure S3. Flow cytometry was performed to detect the expression of PD1, TIM3, and LAG3 in 7D12-T cells and 7D12-T-hIL21 cells.

## Abbreviations

The following abbreviations are used in this manuscript:

ESCC	esophageal squamous cell carcinoma
EGFR	epidermal growth factor receptor
VHHs	variable domain of llama heavy chain of heavy-chain antibodies
Nbs	nanobodies
ScFv	single-chain variable fragment
TNF-α	tumor necrosis factor alpha
IL-2	Interleukin-2
IL-21	Interleukin-21
IFN-γ	interferon-gamma
VEGFR2	vascular endothelial growth factor receptor 2
BCMA	B cell maturation antigen
HER2	human epidermal growth factor receptor 2
TAG-72	tumor-associated glycoprotein 72
PSMA	prostate-specific membrane antigen
MUC1	mucin 1
MM	multiple myeloma
CR	complete response
OR	overall response
UALCAN	University of Alabama at Birmingham Cancer Database
