# Supplementary figures and images for: Interleukin 21-Armed EGFR-VHH-CAR-T Cell Therapy for the Treatment of Esophageal Squamous Cell Carcinoma
Source: Biomedicines. 2025 Jun 30;13(7):1598. doi: 10.3390/biomedicines13071598 (PMC12292304; doi:10.3390/biomedicines13071598)

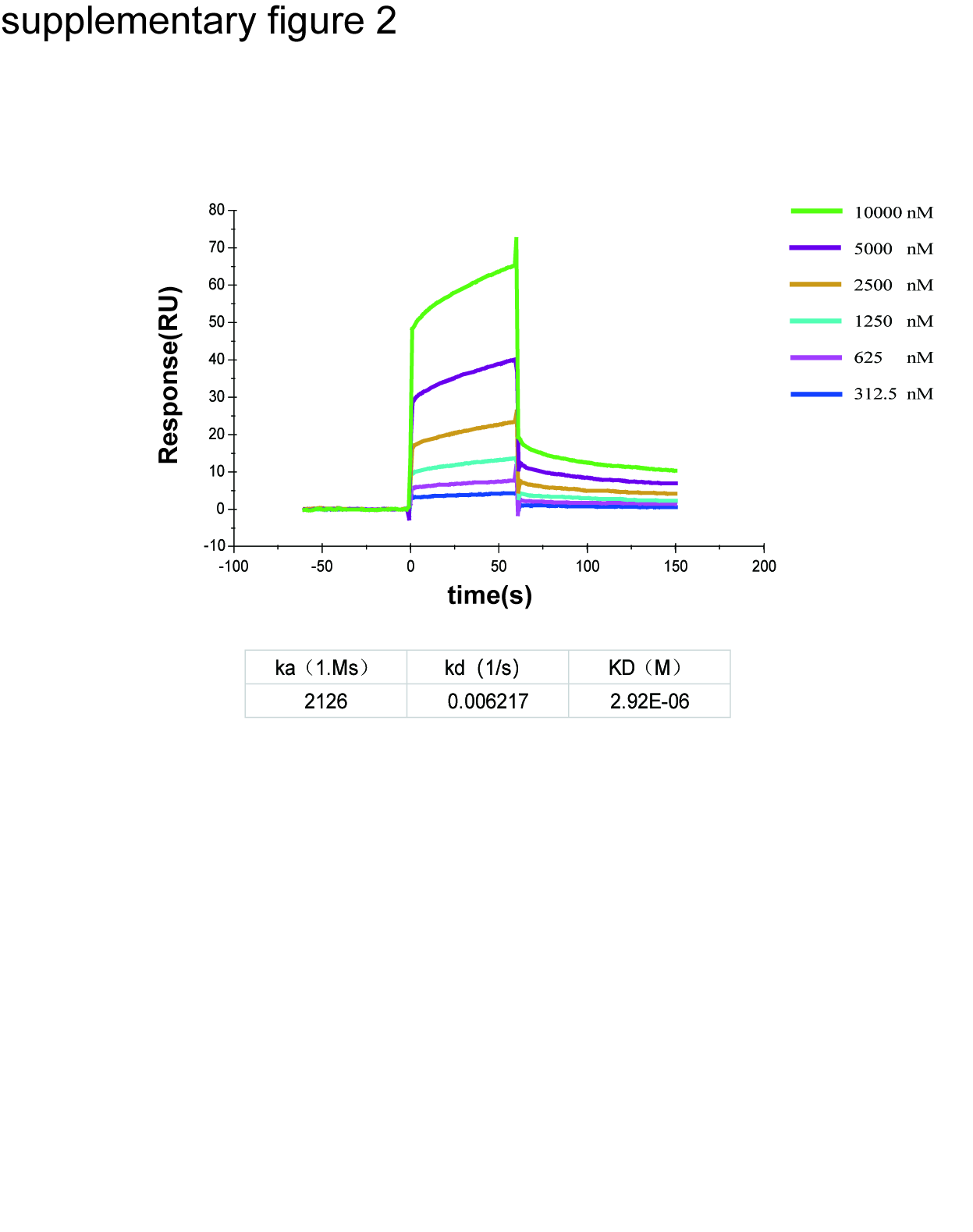

Supplement: Supplementary file 1 [file biomedicines-13-01598-s001.zip › supplement figure s2.tif]

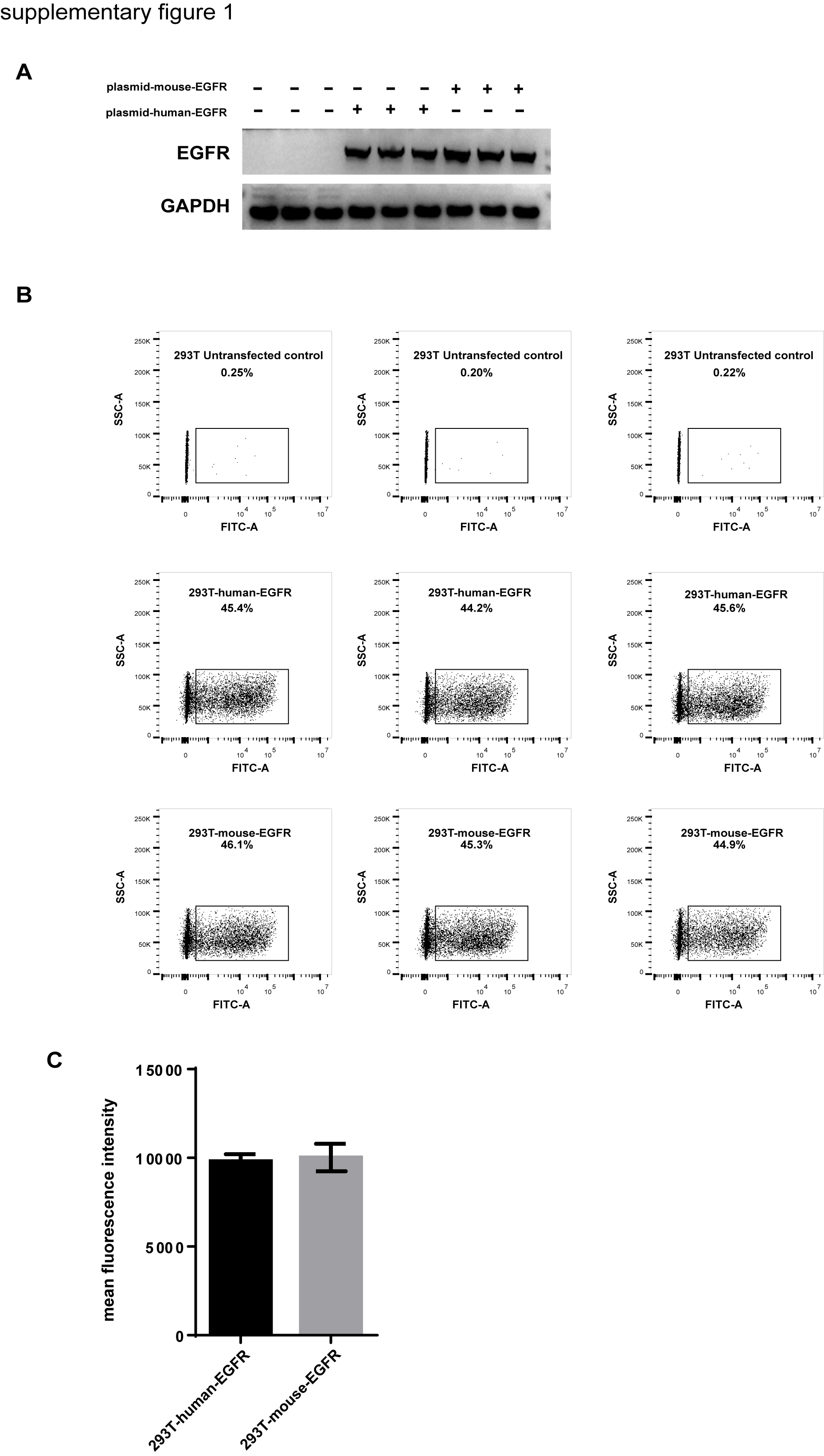

Supplement: Supplementary file 1 [file biomedicines-13-01598-s001.zip › Supplemental figure s1.tif]

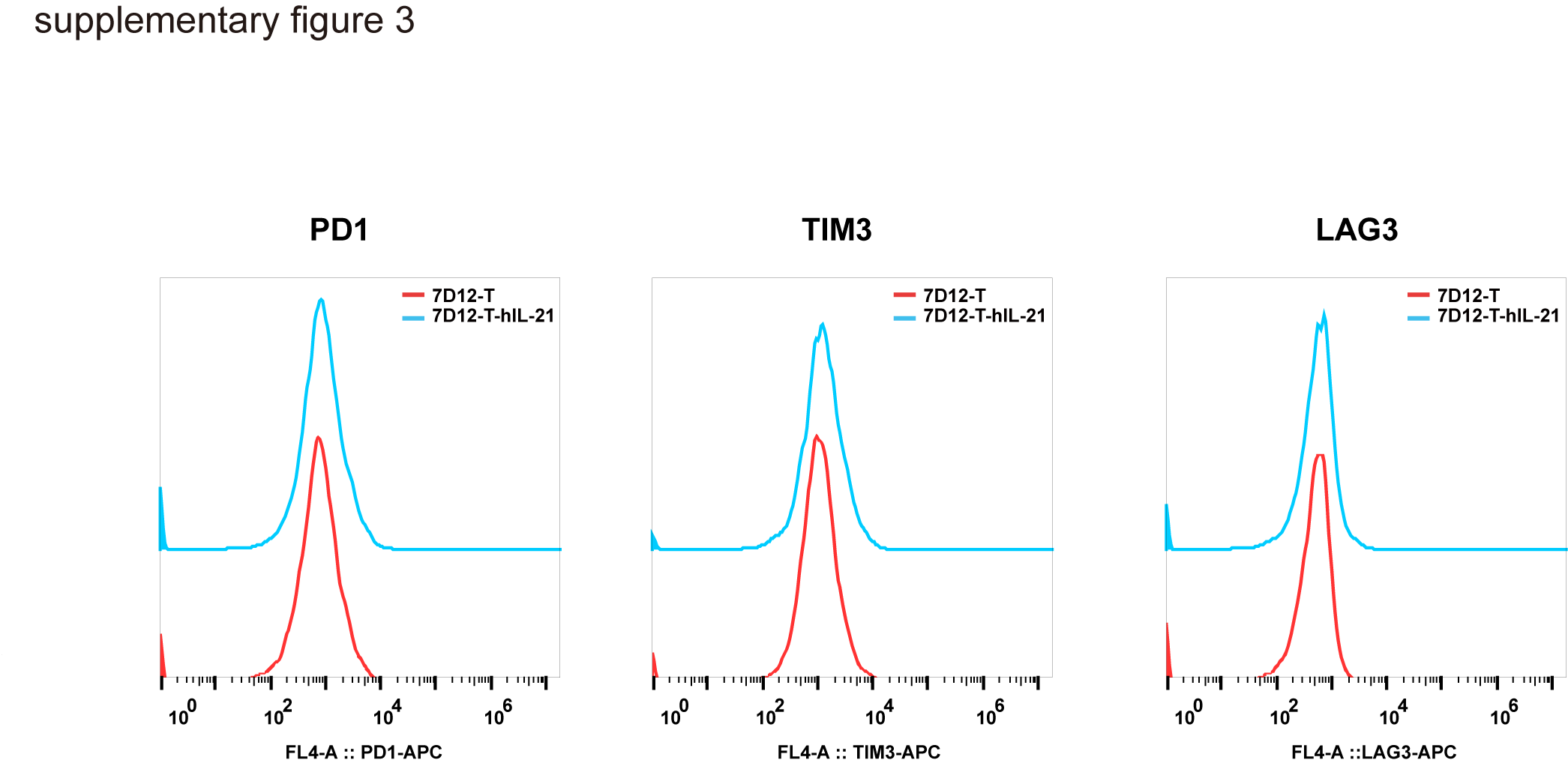

Supplement: Supplementary file 1 [file biomedicines-13-01598-s001.zip › supplemental figure s3.tif]
